# Multi-Omics Characterization of a Novel *SSR4* Variant in Congenital Disorders of Glycosylation

**DOI:** 10.3390/metabo15120786

**Published:** 2025-12-08

**Authors:** Nurulamin Abu Bakar, Nurul Izzati Hamzan, Elyssa Milus Majawit, Siti Nurwani Ahmad Ridzuan, Noor Hafizah Hassan, Anasufiza Habib, Lock-Hock Ngu

**Affiliations:** 1 Centre of Diagnostics, Therapeutics and Investigative Studies (CODTIS), Faculty of Health Sciences, Universiti Kebangsaan Malaysia, Jalan Raja Muda Abdul Aziz, Kuala Lumpur 50300, Malaysia; 2 Special Protein Unit, Specialized Diagnostic Centre, Institute for Medical Research, National Institutes of Health, Jalan Pahang, Kuala Lumpur 50588, Malaysia; nurulizzati.h@moh.gov.my (N.I.H.); siti.nurwani@moh.gov.my (S.N.A.R.); hafizahh@moh.gov.my (N.H.H.); 3 Department of Paediatric, Sabah Women and Child Hospital (SWACH), Kota Kinabalu 88996, Malaysia; elyssamajawit@moh.gov.my; 4 Specialized Diagnostic Centre, Institute for Medical Research, National Institutes of Health, Jalan Pahang, Kuala Lumpur 50588, Malaysia; anasufiza@moh.gov.my; 5 Department of Genetic, Kuala Lumpur Hospital, Jalan Pahang, Kuala Lumpur 50586, Malaysia

**Keywords:** congenital disorders of glycosylation (CDG), *SSR4*, X-linked, multi-omics, metabolomics, glycoproteomics

## Abstract

**Background**: Congenital disorders of glycosylation (CDG) are rare inborn errors of metabolism with multisystemic manifestations. SSR4-CDG is an ultra-rare X-linked subtype caused by pathogenic variants in *SSR4*, a component of the translocon-associated protein (TRAP) complex essential for protein translocation and N-glycosylation. **Case presentation**: We report a two-year-old Malaysian male presenting with global developmental delay, central hypotonia, microcephaly with complete agenesis of the corpus callosum, recurrent infections, bilateral vesicoureteral reflux, and failure to thrive. Growth parameters (weight, length, and head circumference) were persistently below the expected percentiles, indicating postnatal growth restriction. Initial metabolic and biochemical investigations for global developmental delay were unremarkable, apart from mild hyperammonemia. Transferrin isoform analysis demonstrated a type I CDG pattern, raising suspicion of a glycosylation defect. **Results**: Transferrin glycopeptide LC–MS/MS showed impaired N-glycan occupancy at both glycosylation sites (Asn432 and Asn630), with reduced fully sialylated glycoforms and increased non-glycosylated peptides. Targeted metabolomics using triple quadrupole LC–MS/MS revealed systemic abnormalities, including elevated arginine and phenylalanine, reduced glutamate, increased lysophosphatidylcholine (C24:0-LPC), and generalized depletion of free and acylcarnitines. Whole-exome sequencing identified a novel hemizygous *SSR4* variant (c.98del; *p.Pro33LeufsTer23*) on the X chromosome, predicted to produce a truncated, nonfunctional protein. **Conclusions**: This is the first Malaysian patient with SSR4-CDG, comprehensively characterized using a multi-omics diagnostic workflow. The integration of glycoproteomics, metabolomics, and exome sequencing provided a detailed biochemical fingerprint that expands the clinical, genetic, and metabolic spectrum of SSR4-CDG and demonstrates the diagnostic and translational value of multi-omics approaches in inborn errors of metabolism.

## 1. Introduction

Congenital disorders of glycosylation (CDG) are a group of rare inherited metabolic diseases caused by defects in glycan biosynthesis, processing, and transport. About 200 CDG, caused by 189 gene defects, have been identified to date, reflecting the diversity and complexity of glycosylation pathways. The clinical spectrum of CDG is broad, encompassing developmental delay, hypotonia, seizures, coagulopathy, liver dysfunction, and multisystemic manifestations [1]. Because of this heterogeneity and overlapping features, diagnosis is often delayed or missed, particularly in regions where specialized glyco-analytical testing is limited.

SSR4-CDG (previously designated CDG-Iy) is an X-linked disorder of N-glycosylation caused by pathogenic variants in *SSR4*, which encodes the delta (δ)-subunit of the signal sequence receptor (TRAP) complex in the endoplasmic reticulum (ER). The TRAP complex facilitates co-translational translocation of nascent proteins into the ER lumen, a critical step for N-glycan attachment. Loss of SSR4 function disrupts TRAP assembly, leading to incomplete glycosylation of multiple ER-processed glycoproteins [2]. SSR4-CDG is extremely rare, with fewer than 25 patients reported worldwide since the first description in 2014 [2], followed by expanded clinical series [3,4,5]. Core features include global developmental delay, hypotonia, microcephaly, and structural brain abnormalities, most commonly corpus-callosum dysgenesis or hypoplasia, alongside variable hepatic and systemic involvement [3,4,5].

The first-line biochemical screening for CDG traditionally relies on transferrin isoform analysis, which detects altered sialylation patterns indicative of type I or type II glycosylation defects. However, this conventional approach provides an overall glycosylation profile without resolving site-specific abnormalities or subtle glycan structural changes. Recent advances in clinical glycomics and glycoproteomics now allow for more detailed site-specific glycosylation profiling of serum transferrin [6]. When integrated with metabolomics and lipidomics, these approaches provide a systems-level understanding of glycosylation disorders, uncovering secondary metabolic perturbations that may serve as disease biomarkers or therapeutic targets.

Here, we report a novel hemizygous *SSR4* truncating variant in a Malaysian patient, comprehensively characterized using a multi-omics diagnostic workflow that integrates transferrin glycopeptide LC–MS/MS, targeted metabolomics, and whole-exome sequencing. This case demonstrates the diagnostic and translational value of combining multi-omics strategies for rare congenital disorders of glycosylation and expands the molecular and biochemical spectrum of SSR4-CDG within the Southeast Asian population.

## 2. Case Presentation

The patient is a two-year-old Malaysian male, the second child of non-consanguineous parents, with one healthy older sibling. He was delivered at 38 weeks of gestation via elective Caesarean section following a previous lower-segment Caesarean. Antenatal ultrasound raised concerns of ventriculomegaly. At birth, he was vigorous, with Apgar scores of 9 and 10 at one and five minutes, respectively. However, at four hours of life, he developed respiratory distress consistent with transient tachypnoea of the newborn. His neonatal course was complicated by bilateral spontaneous pneumothoraces at 18 h of life, requiring intubation and emergency thoracentesis. He was successfully extubated on day four.

At birth, his growth parameters were at the lower percentiles, with a weight of 2.57 kg, length of 45 cm (3rd percentile), and head circumference of 33 cm (15th percentile). Physical examination revealed subtle dysmorphic features, including micrognathia, low-set ears, a short neck with redundant skin folds, and bilateral positional talipes equinovarus. Cardiac evaluation on day two identified a small patent ductus arteriosus.

**Figure 1:**
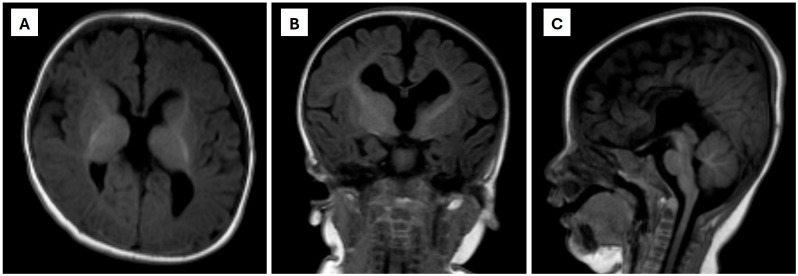
Brain and pituitary magnetic resonance imaging (MRI) findings. T1-weighted axial (**A**), coronal (**B**), and sagittal (**C**) MRI brain images obtained at six months of age show complete agenesis of the corpus callosum, characterized by parallel orientation of the lateral ventricles and colpocephaly. The pituitary gland appears normal on sagittal view.

A cranial ultrasound performed during outpatient follow-up revealed features suggestive of corpus callosum dysgenesis. Subsequent T1-weighted magnetic resonance imaging (MRI) of the brain and pituitary, performed at six months of age, confirmed complete agenesis of the corpus callosum, characterized by parallel orientation of the lateral ventricles and colpocephaly. The pituitary gland appeared normal on sagittal imaging (Figure 1).

During the first six months, the patient experienced recurrent urinary tract infections. Renal ultrasound findings were normal, but a micturating cystourethrogram (MCUG) confirmed grade III vesicoureteral reflux. An initial concern of micropenis (stretched length 2.3 cm, width 0.6 cm) prompted endocrinology referral. Baseline hormonal investigations at one month showed normal thyroid, FSH, LH, testosterone, and cortisol levels, and subsequent follow-up demonstrated penile growth within the expected range for age.

**Figure A1:**
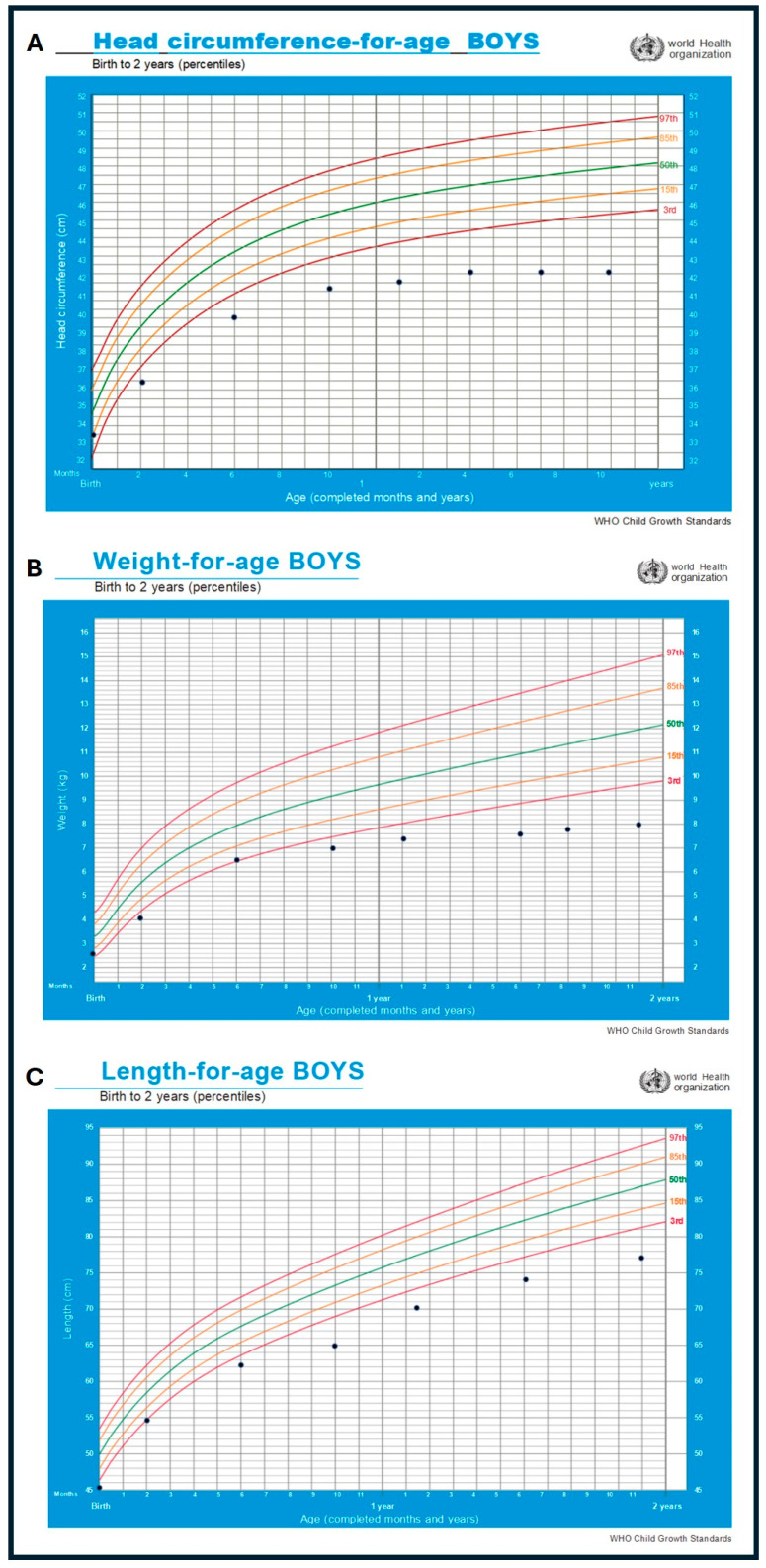
Serial anthropometric growth charts demonstrating decelerating head growth and failure to thrive from birth to 23 months. WHO Child Growth Standards: (**A**) Head circumference-for-age, (**B**) Weight-for-age, and (**C**) Length-for-age, showing all parameters persistently below the 3rd percentile.

Serial growth assessments revealed postnatal microcephaly with decelerating head growth and failure to thrive, with all anthropometric parameters persistently below the expected percentiles (Figure A1). Developmental assessment indicated global developmental delay with central hypotonia. At 24 months of age, his developmental abilities were comparable to those of a 6- to 7-month-old infant. He was able to sit with support and bear weight, grasp and transfer objects, and produce limited vocalizations without meaningful words. Both ophthalmologic and hearing evaluations were within normal limits. Physical examination during follow-up revealed central hypotonia and brisk deep-tendon reflexes, with no hepatosplenomegaly observed. Key clinical and biochemical parameters at diagnosis are summarized in Table A1.

The initial metabolic and biochemical work-up for global developmental delay was largely unremarkable apart from mild hyperammonemia and an isolated amino acid abnormality. Venous blood gas analysis demonstrated normal parameters (pH 7.38, pCO_2_ 40 mmHg, pO_2_ 40 mmHg, HCO_3_^−^ 23.7 mmol/L, BE −1.1). Serum lactate was within the normal range (1.98 mmol/L; reference < 2.2 mmol/L), whereas plasma ammonia was mildly elevated (66.1 µmol/L; reference < 50 µmol/L). Screening for inborn errors of metabolism revealed an isolated elevation of arginine, with otherwise normal amino-acid and acylcarnitine profiles. Plasma amino-acid and urine organic-acid analyses were unremarkable. Thyroid and liver function tests were within normal limits for age, and review of neonatal records showed no documented episodes of hypoglycemia.

Transferrin isoform analysis by capillary electrophoresis demonstrated a type I CDG pattern, with increased disialo-transferrin (5.2%; reference 0.1–1.1) and reduced tetrasialo-transferrin (79.6%; reference 80–90.9) (Table 1). These findings were consistent with impaired N-glycosylation and raised clinical suspicion of a congenital disorder of glycosylation (CDG).

Given the constellation of central nervous system abnormalities, dysmorphic features, multisystem involvement, and biochemical evidence of defective glycosylation, a genetic etiology was strongly suspected. Therefore, whole-exome sequencing (WES) was performed to identify potential pathogenic variants associated with congenital disorders of glycosylation or related metabolic diseases. In parallel, transferrin glycopeptide profiling by Liquid Chromatography-Tandem Mass Spectrometry (LC–MS/MS) was undertaken to characterize site-specific N-glycan occupancy, while targeted metabolomics was applied to explore broader metabolic disturbances. These complementary approaches were designed to provide a comprehensive multi-omics perspective to support diagnosis and uncover secondary biochemical signatures of CDG.

## 3. Materials and Methods

### 3.1. Sample Collection and Ethical Considerations

Peripheral blood samples were obtained from the patient for biochemical, metabolomic, and genetic investigations after informed parental consent. All procedures were conducted in accordance with institutional ethical standards and the Malaysian Good Clinical Practice guidelines. Age-matched healthy pediatric sera (*n* = 5) served as reference comparators for metabolomic and glycopeptide analyses.

### 3.2. Transferrin Isoform Analysis

Serum transferrin isoform analysis was performed using capillary electrophoresis (Sebia CAPILLARYS 2, Lisses, France) at the Institute for Medical Research (IMR), Malaysia. Isoforms were quantified as the percentage of total transferrin and compared against established reference intervals in healthy controls to detect patterns characteristic of congenital disorders of glycosylation (CDG).

### 3.3. Transferrin Glycopeptide LC–MS/MS Analysis

Serum transferrin glycopeptide analysis was performed using an Agilent 6495D Triple Quadrupole LC–MS/MS system with a validated targeted workflow (Arcadia Life Sciences, Kuala Lumpur, Malaysia). Briefly, 5 µL of serum was reduced, alkylated, and digested with sequencing-grade trypsin. The resulting peptides were analyzed by LC–MS/MS in multiple-reaction-monitoring (MRM) mode.

Data acquisition and processing were conducted using Agilent MassHunter version 12 (Agilent Technologies Inc., Santa Clara, CA, USA) and Skyline version 25.1.0.142 (MacCoss Lab, University of Washington, WA, USA) software. Site-specific glycopeptides were annotated according to the glycan composition at the two known N-glycosylation sites of transferrin (Asn432 and Asn630). Statistical significance was determined using two-tailed Student’s *t*-tests (*p* < 0.05).

### 3.4. Targeted Metabolomics Profiling by LC–MS/MS

Targeted serum metabolomics profiling was performed using the NeoBase™ 2 Non-Derivatized MS/MS Kit (Revvity, Turku, Finland) on an Agilent 6495D LC–MS/MS system operating in MRM mode (Arcadia Life Sciences, Kuala Lumpur, Malaysia). The validated method, routinely used for inborn errors of metabolism (IEM) screening, covered amino acids, acylcarnitines, and selected lipid species, including lysophosphatidylcholines (LPCs).

Metabolites were extracted by methanol-based protein precipitation and quantified using isotopically labeled internal standards. Calibration and quantification were based on standard curves, and results were compared with age-matched reference values. Statistical significance was determined using two-tailed Student’s *t*-tests (*p* < 0.05).

### 3.5. Whole-Exome Sequencing (WES)

Whole-exome sequencing (WES) was conducted through a certified clinical sequencing provider (3Billion, Seoul, Republic of Korea). Genomic DNA was extracted from peripheral blood and analyzed using the provider’s validated clinical exome pipeline. Variants were filtered to identify rare, protein-altering changes consistent with the patient’s phenotype. Follow-up segregation testing and functional validation are recommended for definitive molecular confirmation.

## 4. Results

### 4.1. Transferrin Glycopeptide LC-MS/MS

**Figure 2:**
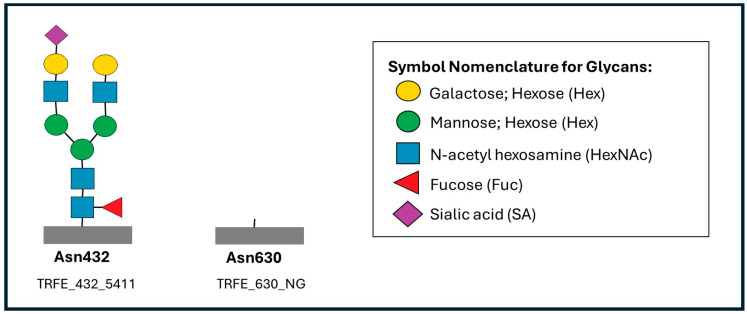
Symbolic representation of the transferrin glycopeptides TRFE_432_5411 and TRFE_630_NG corresponding to the N-glycosylation sites at Asn432 and Asn630, respectively. TRFE_432_5411 represents a glycopeptide with the composition Hex_5_HexNAc_4_Fuc_1_SA_1_, whereas TRFE_630_NG indicates the non-glycosylated peptide. Glycan residues are depicted according to the Symbol Nomenclature for Glycans (SNFG): galactose (yellow circle), mannose (green circle), N-acetylglucosamine (blue square), fucose (red triangle), and sialic acid (purple diamond).

Human serum transferrin (TRFE; UniProt ID: P02787, TRFE_HUMAN) is a major iron-binding transport glycoprotein that carries two N-glycosylation sites, located at Asn432 and Asn630. Site-specific glycosylation was analyzed by tryptic digestion followed by LC–MS/MS of the corresponding glycopeptides. In this nomenclature, the three-digit code following “TRFE” indicates the glycosylation site (432 or 630), while the four-digit code represents the glycoform composition. For example, TRFE_432_5411 corresponds to a glycopeptide containing five hexoses (Hex), four N-acetylhexosamines (HexNAc), one fucose (Fuc), and one sialic acid (SA) at Asn432. Conversely, TRFE_630_NG denotes the non-glycosylated peptide at Asn630 (0 Hex, 0 HexNAc, 0 Fuc, 0 SA) (Figure 2).

Serum transferrin glycopeptide analysis by LC–MS/MS revealed clear site-specific abnormalities at both N-glycosylation sites (Asn432 and Asn630). At Asn432, the sialylated biantennary glycan TRFE_432_5401 was significantly reduced, whereas the fucosylated biantennary glycan TRFE_432_5412, the sialylated triantennary glycan TRFE_432_6503, and the non-glycosylated peptide TRFE_432_NG were elevated (*p* < 0.05). At Asn630, multiple sialylated (TRFE_630_5401, TRFE_630_5402) and fucosylated (TRFE_630_5411, TRFE_630_5412) biantennary glycans were reduced, accompanied by a significant increase in the non-glycosylated peptide TRFE_630_NG (*p* < 0.05). These site-specific abnormalities are summarized in Table 2.

Together, these findings demonstrate a type I glycosylation defect, characterized by the loss of mature sialylated glycans and the accumulation of non-glycosylated transferrin peptides. Notably, the differing patterns between Asn432 and Asn630 indicate site-specific impairment of N-glycan processing, highlighting the diagnostic value of LC–MS/MS-based glycopeptide profiling in detecting subtle glycosylation defects that may not be evident in conventional transferrin isoform electrophoresis

### 4.2. Targeted Metabolomics Profiling

Targeted metabolomics profiling was performed using triple quadrupole LC–MS/MS in multiple reaction monitoring (MRM) mode, covering amino acids, acylcarnitines, and selected lipid species. The patient exhibited significant metabolic abnormalities compared with age-matched controls.

For amino acids, arginine (43.7 µM vs. 29.3 µM, *p* < 0.01) and phenylalanine (31.9 µM vs. 23.9 µM, *p* < 0.05) were elevated, whereas glutamate was reduced (32.2 µM vs. 45.3 µM, *p* < 0.05). In the lipid fraction, lysophosphatidylcholine C24:0-LPC was markedly increased (2.15 µM vs. 0.06 µM, *p* < 0.01). Analysis of carnitine species revealed a pronounced reduction in free carnitine (C0: 2.9 µM vs. 16.1 µM, *p* < 0.01), accompanied by generalized decreases across short-, medium-, and long-chain acylcarnitines (C2, C3, C8–C18). These findings are summarized in Table 3.

Collectively, the targeted metabolomics profile revealed systemic metabolic disturbances characterized by elevated arginine and phenylalanine, reduced glutamate, and markedly decreased levels of free carnitine and acylcarnitines. The generalized depletion of the carnitine pool suggests a secondary impairment of mitochondrial fatty acid oxidation, consistent with energy metabolism dysregulation often observed in congenital disorders of glycosylation. These findings underscore a multisystemic metabolic impact arising from defective glycosylation in SSR4-CDG.

### 4.3. Genetic Analysis

Whole-exome sequencing (WES) was performed through the 3Billion clinical exome sequencing service. Analysis revealed a novel hemizygous frameshift variant in SSR4 (c.98del; p.Pro33LeufsTer23) located on the X chromosome. This single-nucleotide deletion introduces a premature stop codon, predicting a truncated and nonfunctional SSR4 protein.

This variant has not been previously reported in population databases or published literature and was therefore classified as pathogenic. Given the patient’s hemizygous state and consistent clinical and biochemical phenotype, this *SSR4* variant is considered the likely molecular cause of the disorder. However, maternal segregation testing and functional validation are warranted to confirm pathogenicity and establish a definitive molecular diagnosis.

## 5. Discussion

SSR4-CDG is an ultra-rare X-linked disorder of N-glycosylation caused by pathogenic variants in SSR4, which encodes the δ-subunit of the signal sequence receptor (TRAP) complex. Since its first description in 2014 [2], fewer than 25 patients have been reported worldwide [2,3,4,5]. The clinical spectrum typically includes global developmental delay, hypotonia, microcephaly, and brain malformations, often accompanied by multisystem involvement.

Neuroimaging findings in previously reported SSR4-CDG patients have included corpus callosum hypoplasia, cerebral atrophy, and delayed myelination [3,4,5]. In contrast, our patient demonstrated complete agenesis of the corpus callosum, confirmed by MRI, representing one of the most severe structural brain manifestations described to date. This finding expands the known neurological phenotype of SSR4-CDG and underscores the essential role of glycosylation in central nervous system development.

A novel hemizygous *SSR4* variant (c.98del; p.Pro33LeufsTer23) was identified in this patient. This single-nucleotide deletion is predicted to cause premature truncation of the SSR4 protein, resulting in loss of function. To our knowledge, this is the first report of this variant, thereby expanding the mutational spectrum of *SSR4*-CDG. Previous studies have reported both truncating and missense variants [2,3,4,5] associated with similar neurological and systemic phenotypes, supporting a shared pathogenic mechanism. Loss of SSR4 disrupts the TRAP complex required for co-translational translocation of nascent proteins into the endoplasmic reticulum, leading to reduced N-glycan occupancy on multiple glycoproteins and secondary perturbation of cellular metabolism. This mechanistic link provides a plausible explanation for the multi-system phenotype observed in our patient and is consistent with prior work implicating TRAP dysfunction in CDG pathogenesis. While functional validation and segregation analysis were not performed, the molecular, biochemical, and clinical findings collectively support the diagnosis of *SSR4*-CDG.

Biochemical confirmation of CDG traditionally relies on transferrin isoform analysis, which in this case demonstrated a type I CDG pattern. However, this technique provides only global glycosylation profiles and lacks site-specific resolution. Through transferrin glycopeptide LC–MS/MS analysis, we identified differential impairment of N-glycan occupancy at Asn432 and Asn630, characterized by increased non-glycosylated peptides and reduced fully sialylated glycoforms. These site-specific glycosylation changes may represent a disease-specific biochemical signature, underscoring the superior diagnostic sensitivity of glycoproteomics over conventional isoform assays.

In addition, targeted metabolomics profiling revealed secondary metabolic abnormalities. Elevated arginine and phenylalanine, reduced glutamate, and increased C24:0-lysophosphatidylcholine (LPC) suggest dysregulation of amino acid and lipid metabolism, while the generalized reduction in free carnitine and acylcarnitines indicates secondary impairment of mitochondrial fatty acid oxidation. Although mitochondrial dysfunction has been reported as a secondary feature in other CDG subtypes [7], such metabolomic alterations have not previously been described in *SSR4*-CDG. This expands the biochemical phenotype of the disorder and highlights potential avenues for biomarker discovery and metabolic monitoring.

Taken together, our findings demonstrate the diagnostic value of a multi-omics workflow. The integration of genomics, glycoproteomics, and metabolomics provided complementary insights that collectively strengthened the diagnostic conclusion, which was subsequently confirmed by whole-exome sequencing. Importantly, the combined interpretation of glycoproteomic and metabolomic data allowed cross-validation of the biochemical phenotype: site-specific under-glycosylation of transferrin was accompanied by metabolic signatures of impaired energy and amino acid homeostasis, reinforcing the presence of a systemic glycosylation defect linked to the identified SSR4 variant. For ultra-rare inborn errors of metabolism such as SSR4-CDG, comprehensive multi-omics approaches not only improve diagnostic precision but also deepen our understanding of the downstream metabolic consequences of defective glycosylation. This concept aligns with recent perspectives that emphasize the integration of multi-omics platforms for CDG diagnostics and biomarker discovery [6,8,9].

A limitation of this report is the single-patient design and the absence of functional studies to directly assess SSR4 expression or TRAP complex integrity, as well as the lack of maternal carrier testing. Future work should include cellular assays, segregation analysis, and experimental modeling of the c.98del (p.Pro33LeufsTer23) variant, for example using CRISPR-based approaches, together with expanded (including untargeted) metabolomic profiling in additional SSR4-CDG patients to validate disease-specific metabolic signatures and refine potential biomarkers.

## 6. Conclusions

We report the first Malaysian patient with *SSR4*-CDG carrying a novel hemizygous truncating variant (c.98del; p.Pro33LeufsTer23). Beyond conventional transferrin isoform analysis, we applied a multi-omics diagnostic workflow integrating transferrin glycopeptide LC–MS/MS, targeted metabolomics, and whole-exome sequencing. This comprehensive approach confirmed impaired N-glycan occupancy at both transferrin glycosylation sites and revealed secondary metabolic disturbances, including abnormalities in amino acid, lipid, and carnitine metabolism.

Importantly, the presence of complete agenesis of the corpus callosum represents one of the most severe neuroanatomical manifestations reported in SSR4-CDG to date, further extending the known clinical spectrum of this disorder. Collectively, these findings broaden the clinical, genetic, and biochemical landscape of SSR4-CDG and underscore the diagnostic and translational value of multi-omics strategies for improving diagnostic precision and identifying potential biomarkers in inborn errors of metabolism.

## Figures and Tables

**Table 1:** Transferrin isoform analysis by capillary electrophoresis in the SSR4-CDG patient compared with controls.

Transferrin Isoform	Patient (%)	Reference Range (%)	Interpretation
Asialo-transferrin	0.3	0.0–0.2	Slightly elevated
Disialo-transferrin	5.2	0.1–1.1	Increased
Trisialo-transferrin	14.9	6.3–12.4	Mildly elevated
Tetrasialo-transferrin	79.6	80.0–90.9	Reduced
Pentasialo-transferrin	0.0	0.1–1.1	Within normal limits
Hexasialo-transferrin	0.0	0.0–0.3	Absent

**Table 2:** Transferrin glycopeptide LC-MS/MS results in the SSR4-CDG patient compared with controls.

Site	Glycopeptide	Glycoform Composition	* Finding in Patient	Interpretation
Asn432	TRFE_432_5401	Hex5HexNAc4Fuc0SA1	↓ Reduced	Loss of sialylated biantennary glycans (Asn432)
Asn432	TRFE_432_5412	Hex5HexNAc4Fuc1SA2	↑ Elevated	High fucosylated biantennary glycans (Asn432)
Asn432	TRFE_432_6503	Hex6HexNAc5Fuc0SA3	↑ Elevated	High sialylated triantennary glycans (Asn432)
Asn432	TRFE_432_NG	Non-glycosylated peptide	↑ Elevated	Impaired glycan occupancy (Asn 432)
Asn630	TRFE_630_5401	Hex5HexNAc4Fuc0SA1	↓ Reduced	Loss of sialylated biantennary glycans (Asn630)
Asn630	TRFE_630_5402	Hex5HexNAc4Fuc0SA2	↓ Reduced	Loss of sialylated biantennary glycans (Asn630)
Asn630	TRFE_630_5411	Hex5HexNAc4Fuc1SA1	↓ Reduced	Loss of fucosylated biantennary glycans (Asn630)
Asn630	TRFE_630_5412	Hex5HexNAc4Fuc1SA2	↓ Reduced	Loss of fucosylated biantennary glycans (Asn630)
Asn630	TRFE_630_NG	Non-glycosylated peptide	↑ Elevated	Impaired glycan occupancy (Asn630)

* Arrows indicate direction of change in the patient relative to controls. All differences were statistically significant (*p* < 0.05).

**Table 3:** Targeted metabolomics results in the SSR4-CDG patient compared with age-matched controls.

Metabolite Class	Compound	Patient Value (µM)	Control Mean (µM)	* Change
Amino acid	Arginine	43.7	29.3	↑ Elevated (*p* < 0.05)
	Phenylalanine	31.9	23.9	↑ Elevated (*p* < 0.05)
	Glutamate	32.2	45.3	↓ Reduced (*p* < 0.05)
Lipids	C24:0-LPC	2.15	0.06	↑ Markedly elevated (*p* < 0.01)
Carnitines	Free carnitine (C0)	2.9	16.1	↓ Markedly reduced (*p* < 0.01)
	Acylcarnitines (C2, C3, C8–C18)	Generalized reduction	Within normal range	↓ Reduced (*p* < 0.05)

* Arrows indicate direction of change; *p* < 0.05 considered statistically significant.

## Data Availability

The datasets generated and analyzed during the current study are included within this article and its Appendix A. Further inquiries can be directed to the corresponding author.

## Abbreviations

The following abbreviations are used in this manuscript:

CDG	Congenital disorders of glycosylation (CDG)
ER	endoplasmic reticulum
δ	Delta
TRAP	signal sequence receptor
MCUG	micturating cystourethrogram
MRI	magnetic resonance imaging
WES	whole-exome sequencing
LC-MS/MS	Liquid Chromatography-Tandem Mass Spectrometry
TRFE	Human serum transferrin
SNFG	Symbol Nomenclature for Glycans
Asn	Asparagine
NG	non-glycosylated
Hex	Hexose
HexNAc	N-acetyl hexosamine
Fuc	Fucose (Fuc)
SA	Sialic acid
LPC	lysophosphatidylcholine

## Appendix A

**Table A1:** Clinical and biochemical parameters of the SSR4-CDG patient compared with age-matched reference values [10,11,12,13,14].

Parameter/Test	Patient Value/Summary	Age at Test	Reference Range/Comment
Perinatal/growth			
Birth weight	2.57 kg	At birth	2.5–4.0 kg (term male infant)
Head circumference	Below 3rd centile from early infancy; decelerating head growth (microcephaly)	Infancy/follow-up	WHO growth standards for boys 0–2 years
Weight-for-age/length-for-age	Persistently below expected percentiles (failure to thrive)	Infancy/follow-up	WHO growth standards for boys 0–2 years
Basic Biochemistry			
Liver function test	Total bilirubin 27.5 µmol/L; ALT 15 U/L; AST 19 U/L; ALP 248 U/L; albumin 33 g/L; globulin 17 g/L; total protein 58 g/L	1 month	Local pediatric reference ranges
Venous blood gas	pH 7.38; pCO_2_ 40 mmHg; pO_2_ 40 mmHg; HCO_3_^−^ 23.7 mmol/L; BE −1.1	2 months	Local pediatric reference ranges
Ammonia	66.1 µmol/L	11 months	<50 µmol/L (mildly elevated)
Lactate	1.98 mmol/L	16 months	<2.2 mmol/L
Thyroid function test	TSH 0.87 mIU/L; free T4 12.65 pmol/L	16 months	Within reference ranges [10]
Endocrine/genital			
Penile length (stretched)	2.3 cm at 1 month; 3.6 cm at 13 months	Infancy/follow-up	Within reported Asian neonatal reference range for term males [11,12]
Gonadotropins/sex steroids (FSH, LH, testosterone)	Within age-appropriate reference ranges	1 month	Based on published pediatric endocrine reference intervals [13]
Cortisol	Within age-appropriate reference range	1 month	Within reference ranges [14]
Inborn errors of metabolism screening			
Newborn IEM screening (DBS amino acids and acylcarnitines)	Isolated mild increase in arginine; other amino acids and acylcarnitines normal	11 months	Local pediatric reference ranges
Plasma amino acid profile	All amino acids within normal limits		Local pediatric reference ranges
Urine organic acid profile	No significant abnormal peaks		Local pediatric reference ranges
